# Elucidating the dynamic immune responses within the ocular mucosa of rainbow trout (*Oncorhynchus mykiss*) after infection with *Flavobacterium columnare*

**DOI:** 10.3389/fimmu.2023.1288223

**Published:** 2023-11-21

**Authors:** Weiguang Kong, Peng Yang, Guangyi Ding, Gaofeng Cheng, Zhen Xu

**Affiliations:** ^1^Key Laboratory of Breeding Biotechnology and Sustainable Aquaculture, Institute of Hydrobiology, Chinese Academy of Sciences, Wuhan, China; ^2^Department of Aquatic Animal Medicine, College of Fisheries, Huazhong Agricultural University, Wuhan, Hubei, China

**Keywords:** ocular mucosa, mucosal immune response, RNA-Seq, *F. columnare*, *Oncorhynchus mykiss*

## Abstract

The eye of vertebrates is constantly faced with numerous challenges from aquatic or airborne pathogens. As a crucial first line of defense, the ocular mucosa (OM) protects the visual organ from external threats in vertebrates such as birds and mammals. However, the understanding of ocular mucosal immunity in early vertebrates, such as teleost fish, remains limited, particularly concerning their resistance to bacterial infections. To gain insights into the pivotal role of the OM in antibacterial immunity among teleost fish, we developed a bacterial infection model using *Flavobacterium columnare* in rainbow trout (*Oncorhynchus mykiss*). Here the qPCR and immunofluorescence results showed that *F. columnare* could invade trout OM, suggesting that the OM could be a primary target and barrier for the bacteria. Moreover, immune-related genes (*il-6*, *il-8*, *il-11*, *cxcl10*, *nod1*, *il1-b*, *igm*, *igt*, etc.) were upregulated in the OM of trout following *F. columnare* infection, as confirmed by qPCR, which was further proved through RNA-seq. The results of transcriptome analyses showed that bacterial infection critically triggers a robust immune response, including innate, and adaptive immune-related signaling pathways such as Toll-like, NOD-like, and C-type lectin receptor signaling pathway and immune network for IgA production, which underscores the immune role of the OM in bacterial infection. Interestingly, a substantial reduction in the expression of genes associated with visual function was observed after infection, indicating that bacterial infection could impact ocular function. Overall, our findings have unveiled a robust mucosal immune response to bacterial infection in the teleost OM for the first time, providing valuable insights for future research into the mechanisms and functions of ocular mucosal immunity in early vertebrate species.

## Introduction

The eye is a specialized organ as a sensory transmitter for visual signals. It is the primary instrument for the vertebrate to carry out crucial tasks including socialization, survival, migration, and reproductive selection (1). As the eye performs function, it is continuously exposed to the external environment with the risk of external pathogen invasion, such as bacteria, parasites, and viruses (2). To effectively combat these threats in the environment, vertebrate eyes evolved a diverse repertoire of strategies to protect visual function, in which the ocular mucosa (OM) immune system plays an essential role. In higher vertebrates, the OM consists of the lacrimal gland, conjunctiva, and cornea (3), which constitute the OM system that is specialized to meet the unique needs of the eye such as lubrication and host defense (4). While the structure of the eye is relatively simple in ancient vertebrates such as teleost, specifically, their OM lacks the protection of eyelids and eyelashes (5). This means that the OM of teleost may face a more difficult challenge in a pathogen-infested aquatic environment. Therefore, we hypothesized that the OM of teleost fish plays a more important part in immune defense.

The interaction between the OM immune system and pathogens has been extensively studied in vertebrates. Previous studies have indicated that OM serves as an entry site for pathogen infections, such as parasites (6), viruses (7), and bacteria (8). As a result, the OM can activate appropriate innate or adaptive immune responses to pathogen invasion. In mammals, some immune cells harbored in the OM could mount a selective response to bacterial components and then induce limited inflammation—for instance, macrophages can recognize different bacteria and induce inflammation *via* Toll-like receptors to eliminate pathogens (9). Furthermore, immune-related proteins were upregulated in response to bacterial antigens in the OM of healthy mice and efficiently cleared opportunistic pathogens in a time-dependent manner (10). Recently, a report showed that a bacterial eye disease caused by *Mycoplasma gallisepticum* in house finches (*Haemorhous mexicanus*) suggested that avian OM could activate an extensive immune response against microbial infection (11). The immune functions of OM in birds and mammals have been extensively studied (12, 13), whereas few studies describe the immune response to bacterial infection within OM of teleost fish.

The rainbow trout (*Oncorhynchus mykiss*) is an economically important cold-water species, and it is also an experimental subject for scientific research (14, 15). In recent years, due to the intensification of trout farming, the incidence of infectious disease outbreaks in rainbow trout farms has increased steadily (16), among which columnaris diseases have caused considerable economic losses (17). Columnaris diseases, caused by the Gram-negative bacterium *F. columnare*, seriously affect freshwater hatchery-reared and wild fish populations (18). Typically, *F. columnare* infects fish mucosal tissues, including the gills, skin, and scales, causing serious histological damage (19, 20). Although previous studies have shown that *F. columnare* invasion induces strong immune responses in rainbow trout skin, nose, oropharyngeal mucosa, and gills (21–24), little is known about the effects of *F. columnare* infection on trout OM.

To understand the immune responses in the ancient teleost OM upon bacterial infection, rainbow trout were infected with *F. columnare* by bath. In this study, we found that the bacteria could successfully invade the OM and elicit a strong immune response. Furthermore, a transcriptome analysis revealed that innate and adaptive immunity-related processes occurred in the OM. These findings showed the dynamics of immune response in trout OM after *F. columnare* infection, highlighting the specificity of OM immunity following bacterial invasion. Overall, our findings revealed for the first time the interaction of the OM immune system with bacteria, providing a theoretical basis for understanding mucosal immunity in teleost fish.

## Materials and methods

### Fish maintenance

Rainbow trout (3–5 g) were obtained from a fish farm in Shiyan (Hubei, China). The fish were transported to the Institute of Hydrobiology and kept in aerated aquariums with a water recirculation system, including internal biofilters and thermostatic control. The fish were acclimatized for at least 2 weeks at 16°C and fed commercial trout pellets daily at 1% biomass throughout the experiment.

### *F. columnare* culture and challenge

The green fluorescent protein (GFP)-labeled *F. columnare* G4 strain used in this study was provided by Professor Pin Nie’s lab in the Institute of Hydrobiology, Chinese Academy of Sciences. The construction method of the GFP*-F. columnare* G4 strain was described previously (21). The methods for *F. columnare* G4 strain culture and infection experiment were slightly modified from previous studies (21–24). Briefly, *F. columnare* G4 strain was cultured in Shieh broth plus tobramycin at 28°C for 12–16 h, until OD_600 = _0.4–0.6. A total of 240 healthy rainbow trout were utilized for the purpose of this study. In the experimental group, a total of 120 fish were challenged by immersing with F. columnare G4 at a final concentration of 1 × 106 CFU/mL at 16°C for 4 h and then transferred to the tank which contained clean circulating water. Initially, 42 fish were sacrificed to collect tissue samples at 0.5, 1, 4, 7, 14, 21, and 28 days post infection (DPI). An additional 78 fish were monitored daily to calculate the mortality rate. In the control group, another 120 fish were exposed to the same culture medium without F. columnare and then moved to similar tanks. The OM, spleen (SP), head kidney (HK), skin (SK), and gill (GI) samples were collected at 0.5, 1, 4, 7, 14, 21, and 28 DPI. To collect OM tissue from rainbow trout, make precise cuts along the tissue and meticulously remove the eyeball and underlying muscle tissue beneath the mucosal layer using anatomical scissors.

### Detection of *F. columnare* in rainbow trout after infection

To understand the load of *F. columnare* in different tissues after the challenge, different tissue samples including SP, HK, OM, SK, and GI were collected and homogenized in tissue lysis buffer using steel beads and shaking (60 Hz for 1 min) by TissueLyser II (Jingxin Technology). Total genomic DNA was isolated from SP, HK, OM, SK, and GI using QIAamp DNA Mini Kit (Qiagen). Absolute qPCR analysis was conducted to estimate the copy numbers of *F. columnare* using specific primers as shown in **Supplementary Table S1**. qPCR was conducted in a 20-μL reaction mixture containing 10 μL 2 × SYBR Green qPCR Mix (YEASEN), 0.5 μL of each primer (10 μM), 1.0 μL of 100 ng DNA templates, and 8.0 μL of nuclease-free water. The qPCR program consisted of an initial step at 95°C for 5 min, followed by 40 cycles at 95°C for 10 s and 58°C for 30 s. All samples were analyzed in triplicate to ensure accuracy.

To enhance the detection of *F. columnare* in rainbow trout after infection, PCR amplification was performed on the DNA from selected tissues using the 16S rRNA specific primers listed in **Supplementary Table S1**, as mentioned in a previous study (25). The PCR program was as follows: denaturation at 95°C for 30 s, annealing at 59°C for 30 s, and extension at 72°C for 1 min. A total of 30 cycles were followed by a final extension of 6 min at 72°C. The PCR products were extracted from 2% agarose gel, and images were acquired using Gel Doc XR+ (Bio-Rad, USA). Moreover, OM tissue samples were collected from both control and infected fish and homogenized in 1 mL phosphate-buffered saline to coat the plates, and colonies were observed using a fluorescence microscope. For the detection of *F. columnare* in the trout OM, the ocular tissues of rainbow trout were fixed overnight at 4°C in 4% neutral-buffered formalin. After fixation, the tissues were dehydrated in graded ethanol, embedded in paraffin, and sectioned into 5-μm slices. The sections were stained with DAPI (Invitrogen) for 8 min. All sections were observed under an Olympus BX53 microscope and captured with the CellSense Dimension software.

### RNA isolation and qPCR analysis

Before sampling, the rainbow trout were anesthetized with MS-222 (Sigma, USA) at a concentration of 100 mg/L. Each fish was dissected to obtain OM, SP, HK, SK, and GI samples. Total RNA was extracted from the tissues using Trizol Reagent (Invitrogen) following the manufacturer’s protocol. The integrity of extracted RNA was detected in 1% agarose gel electrophoresis (Agilent Bioanalyser, 2100), and the quantification and concentration were determined by spectrophotometry (Nanodrop ND1000, Thermo Scientific). Then, the total RNA (1 μg) was used immediately for cDNA synthesis with the SuperScript first-strand synthesis system for qPCR (Abcam, Canada). The synthesized cDNA was diluted four times and then used as a template for qPCR. All samples were performed in the following conditions: 95°C for 5 min, followed by 40 cycles at 95°C for 10 s and 58°C for 30 s. Relative mRNA abundances were calculated using the 2^-ΔΔCt^ method (26) and normalized to EF1α. The primers used for qPCR are provided in **Supplementary Table S1**.

### RNA-seq library construction, sequencing, and data analyses

The trout OM samples from the control group and the *F. columnare*-infected group at 1 and 14 DPI were sent to Majorbio Technology Co., Ltd. (Wuhan, China). Briefly, total RNA was extracted using Trizol reagent (Invitrogen, USA), and stranded RNA sequencing library preparation was performed using KCTM Stranded mRNA Library Preparation Kit (Illumina^®^) following the manufacturer’s instructions. The PCR products ranging in length from 200 to 500 bp were enriched, quantified, and finally sequenced on a HiSeq X Ten sequencer (Illumina^®^). The reads were mapped to the rainbow trout genome using STAR (version 2.5.3a) with default parameters (27). The mapped reads were counted by feature (Subread-1.5.1; Bioconductor) (28). Differentially expressed genes (DEGs)were estimated by the edgeR package (version 3.12.1) (29). The low-expressed genes (counters per million <1 in three or more samples) were excluded from the downstream analysis. DEGs were considered if false discovery rate (FDR) ≤ 0.05 and |log_2_ (fold change)| ≥ 1. To further analyze the DEGs, we performed Gene Ontology (GO) enrichment analysis and Kyoto Encyclopedia of Genes and Genomes (KEGG) enrichment using KOBAS (version: 2.1.1) (30).

### Validation of RNA-seq by qPCR

A total of 10 DEGs (five were upregulated and five were downregulated) were randomly selected for qPCR validation. The detection was performed in triplicate for each biological replicate. The relative expression values of the selected genes were calculated using the 2^-ΔΔCt^ method and normalized against the expression levels of the EF1α gene.

### Statistical analysis

Data are presented as mean ± standard deviation. Statistical analysis was performed using SPSS software (version 18.0), and an unpaired Student’s *t*-test was used to assess whether the means were significantly different (*p* < 0.05).

## Result

### Establishment of a model of rainbow trout infected by *F. columnare*

**Figure 1:**
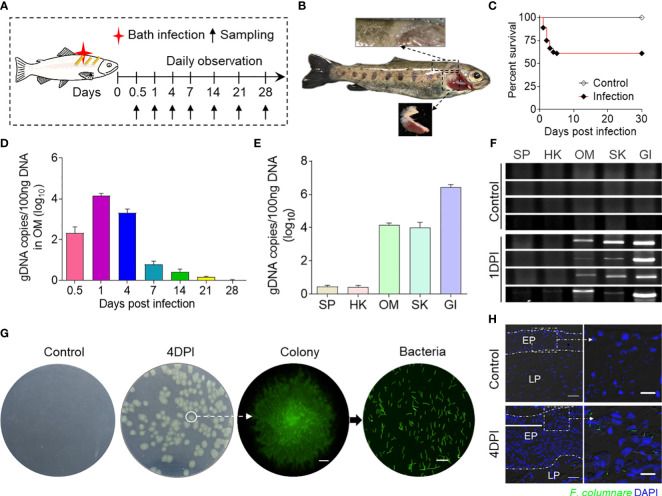
Successful invasion of *F. columnare* into rainbow trout after bath infection. **(A)** A schematic diagram about the timing of infection and sampling. **(B)** Fish mortalities and clinical symptoms were recorded daily for 30 days after *F. columnare* infection. Symptoms of fin rot and gill necrosis appeared in the trout at 4 to 7 days post-infection (DPI). **(C)** Cumulative survival rates of the control and *F. columnare*-infected group. Group comparisons for survival data were made by log-rank test statistics. **(D)** *F. columnare*-gDNA copies (log10) at different time points were assessed in ocular mucosa (OM) samples using a qPCR assay (*n* = 6 fish per group). Data are representative of three independent experiments (mean ± SEM). **(E)** *F. columnare*-gDNA copies (log10) at 1 DPI were assessed in different samples using a qPCR assay (*n* = 6 fish per group). Data are representative of three independent experiments (mean ± SEM). **(F)** The PCR products of the *F. columnare* 16S rRNA gene of the SP, HK, OM, SK, and GI tissues from control and infected fish at 1 DPI were electrophoresed in 2% agarose, and the bands were recorded using the gel imaging system (*n* = 4 fish per group). **(G)** The culture plates from trout BM of control fish and infected fish at 4 DPI. Colony image: a magnified view of circled colony from infected fish by fluorescence microscope (original magnification, ×10), Scale bar, 200 μm. Bacteria image: the observation of bacterial solution obtained by circled colony expansion (original magnification, ×40; scale bar, 10 μm). **(H)** Localization of GFP-*F. columnare* in trout OM of control fish and infected fish at control and 4 DPI. Differential interference contrast images showing merged staining with *F. columnare* (green) and nuclei (blue). EP, epidermis; LP, lamina propria. Scale bars, 100 μm. Scale bar of the enlarged image, 20 μm.

In this study, we developed a bath infection model using *F. columnare* to explore the role of the OM in bacterial infection (**Figure 1A**). During infection, clinical symptoms showed the phenotype of the GI filaments, and the SK became white and rotten, and the amount of mucus was increased (**Figure 1B**). The cumulative mortality rate of the trout in the 30 days of the infection was 38.9%, and the death rate tended to be stable at 7 DPI (**Figure 1C**). Then, we examined the abundance of *F. columnare* in the OM at different time points. The *F. columnare* genome DNA copies peaked at 1 DPI and declined significantly between 14 and 28 DPI (**Figure 1D**). Thus, the amount of *F. columnare* in each tissue was detected at 1 DPI, and the qPCR and PCR results showed that the amount of *F. columnare* is relatively high in the OM and SK, with the highest amount in the GI (**Figures 1E, F**). Importantly, the *F. columnare* load in the OM was comparable to those of other mucosal tissues, one of the target organs of *F. columnare*, which indicated that OM could be considered as an invasion site for *F. columnare.* In addition, the tissue homogenates of trout OM from control fish and 4-DPI fish were cultured on Shieh agar, and the yellow-green color bacterial colonies showed characteristic rhizoid shapes that were detected only from the homogenate samples of infected trout OM. Moreover, the single colonies isolated from the plate were grown in pure culture and displayed characteristic elongated rod-shaped *F. columnare* bacteria with green fluorescence (**Figure 1G**). The results of immunofluorescence showed that fluorescent *F. columnare* could be detected in the epidermal layer of the OM, but not in the control group (**Figure 1H**).

### Immune response in the trout OM with *F. columnare* infection

**Figure 2:**
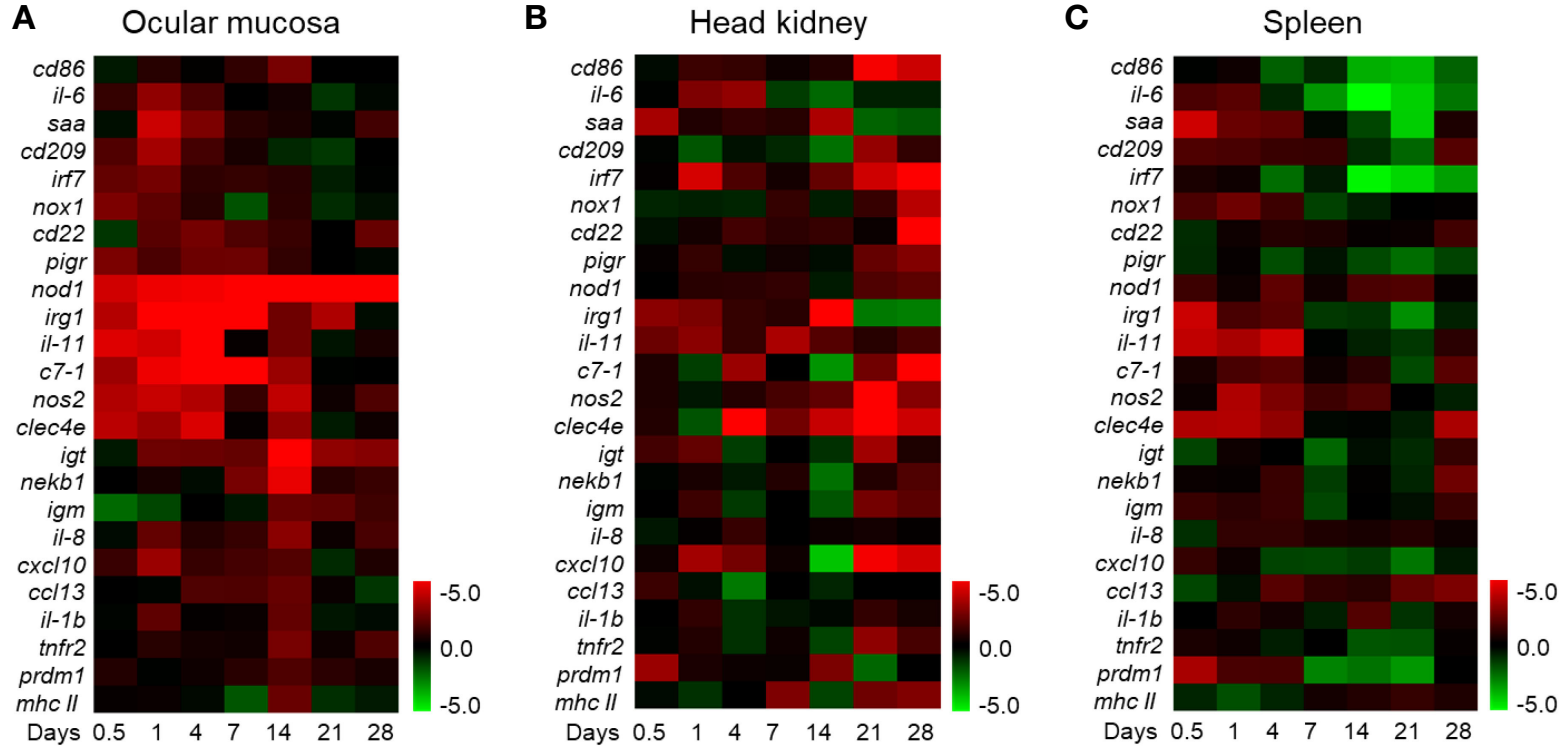
Heat map of immune marker expression in rainbow trout. **(A)** The heat map illustrates the quantitative qPCR results of relative mRNA levels for the selected immune markers in *F. columnare*-infected fish *versus* control fish measured at 0.5, 1, 4, 7, 14, 21, and 28 days post-infection (*n* = 6 fish per group) in the ocular mucosa of rainbow trout. **(B)** Heat map in the head kidney. **(C)** Heat map in the spleen. The color value represents fold change. Data are expressed as mean fold increase in expression. The color represents the relative expression of the corresponding genus, with red indicating the higher expression and green indicating the lower expression.

To further investigate the immune functions of the OM during *F. columnare* infection, we analyzed the mRNA expression patterns of immune-related genes. Remarkably, the qPCR results revealed vigorous immune responses in the OM as well as other immune organs (head kidney and spleen). At 1 DPI, innate immune-related genes such as *saa*, *cd209*, interferon regulatory factor 7, *nox1*, *cxcl10*, *irg1*, *il-6*, *il-1b*, *il-11*, *c7-1*, *nos2*, and *clec4e* exhibited high expression levels in the OM at 1 DPI. Adaptive immune-related genes, such as *cd86*, *cd22*, *nekb1*, immunoglobulin T, immunoglobulin M, *ccl13*, *tnfr2*, *prdm1*, and *mhc Ⅱ*, were upregulated at 14 DPI. Otherwise, various immune genes respond in the OM earlier than the internal immune organs (head kidney and spleen), and the overall immune response intensity of the OM was higher than that of the other two tissues (**Figures 2A–C**). These results suggest that bacteria can stimulate a local mucosal immune response in the OM. Notably, 1 and 14 DPI were the most relevant in terms of the intensity of the immune response in the OM. Therefore, these time points were selected for the subsequent RNA-seq analysis.

### Differentially expressed genes’ functional enrichment analysis

**Figure 3:**
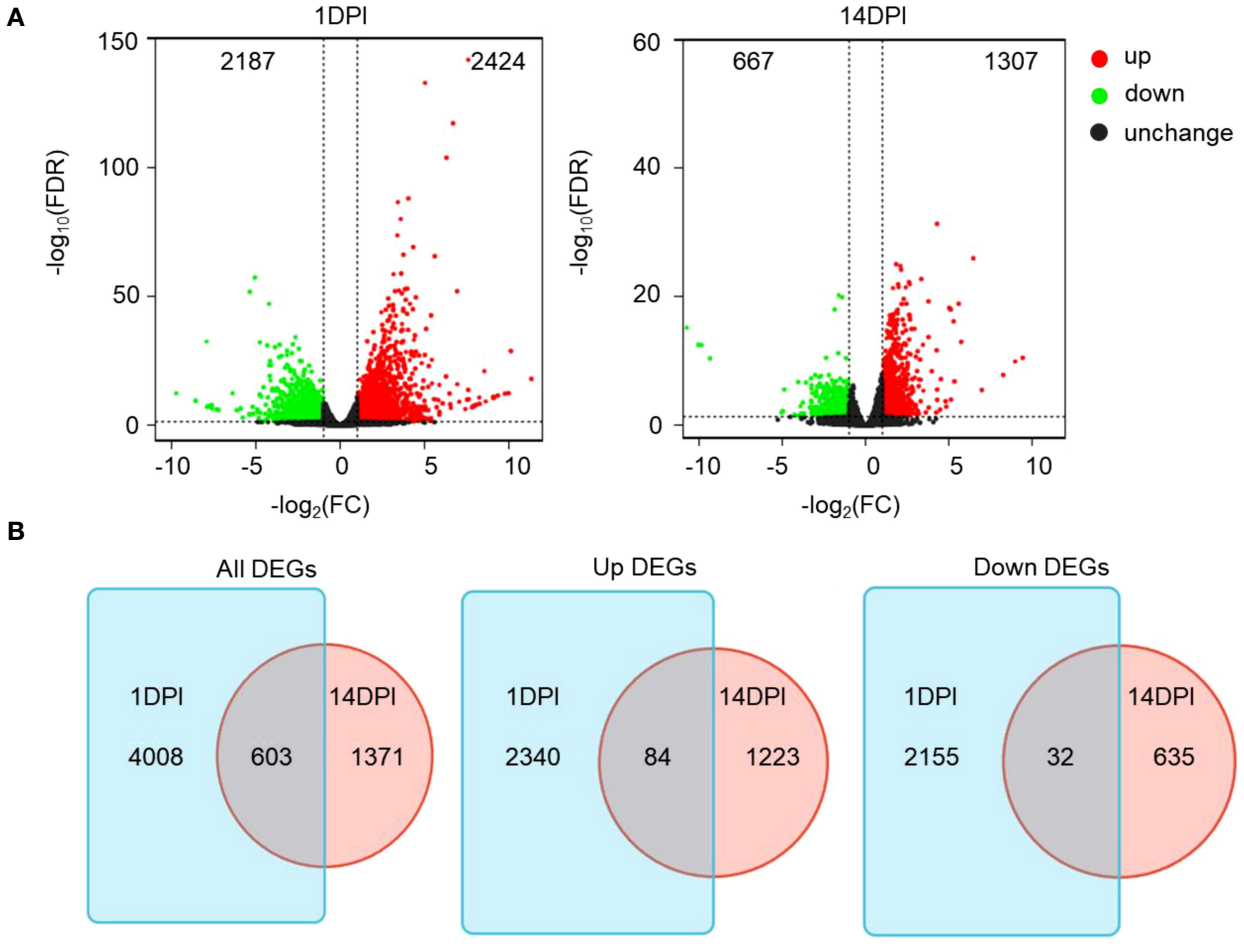
Immune response kinetics were analyzed from the perspective of trout ocular mucosa (OM) transcriptome after infection with *F. columnare* (*n* = 9 fish per group). **(A)** Volcano plots showing the DEGs distribution of OM in trout at 1 DPI (left) and 14 DPI (right) compared to controls. Red spots, expression fold change >2, FDR <0.05. Green spots, expression fold change <2, FDR <0.05. Black spots indicate no difference. The vertical axis represents-log10(FDR), and the horizontal axis represents log_2_ (fold change). **(B)** Venn diagram of RNA-seq experiments showing overlap of up or downregulated genes in rainbow trout OM at 1 or 14 DPI compared to control fish.

Here 12 samples from the two time points mentioned above were divided into four groups (FCC1d, FCE1d, FCC14d, and FCE14d) for transcriptome sequencing. A total of 367,531,421 high-quality clean data were obtained after a series of quality controls, and the percentage of Q30 base in all samples was no less than 90.49% (**Supplementary Table S2**). Further comparison of transcriptome data with reference genome sequences showed that the efficiency of comparison between reads and the reference genome of each sample ranged from 72.46% to 74.39%, and the unique mapped reads ranged from 66.57% to 67.56% (**Supplementary Table S3**). DESeq2 was used for differential expression analysis between sample groups to obtain differentially expressed gene sets between two biological conditions. Fold change represents the ratio of expression between two samples (groups). The FDR is obtained by correcting for the difference of significance *p*-value. Fold change > 2 and FDR < 0.05 were used as the screening criteria. The RNA-seq analysis revealed significant changes in a total of 4,611 genes at 1 DPI and 1,974 genes at 14 DPI following *F. columnare* infection compared to the controls. Among these genes, 2,187 and 667 were upregulated, whereas 2,424, and 1,307 were downregulated at 1 and 14 DPI, respectively (**Figure 3A**). Thus, a total of 603 DEGs existed both in 1 and 14 DPI groups, 84 were upregulated, and 32 were downregulated (**Figure 3B**).

### Functional enrichment analysis of differentially expressed genes

**Figure 4:**
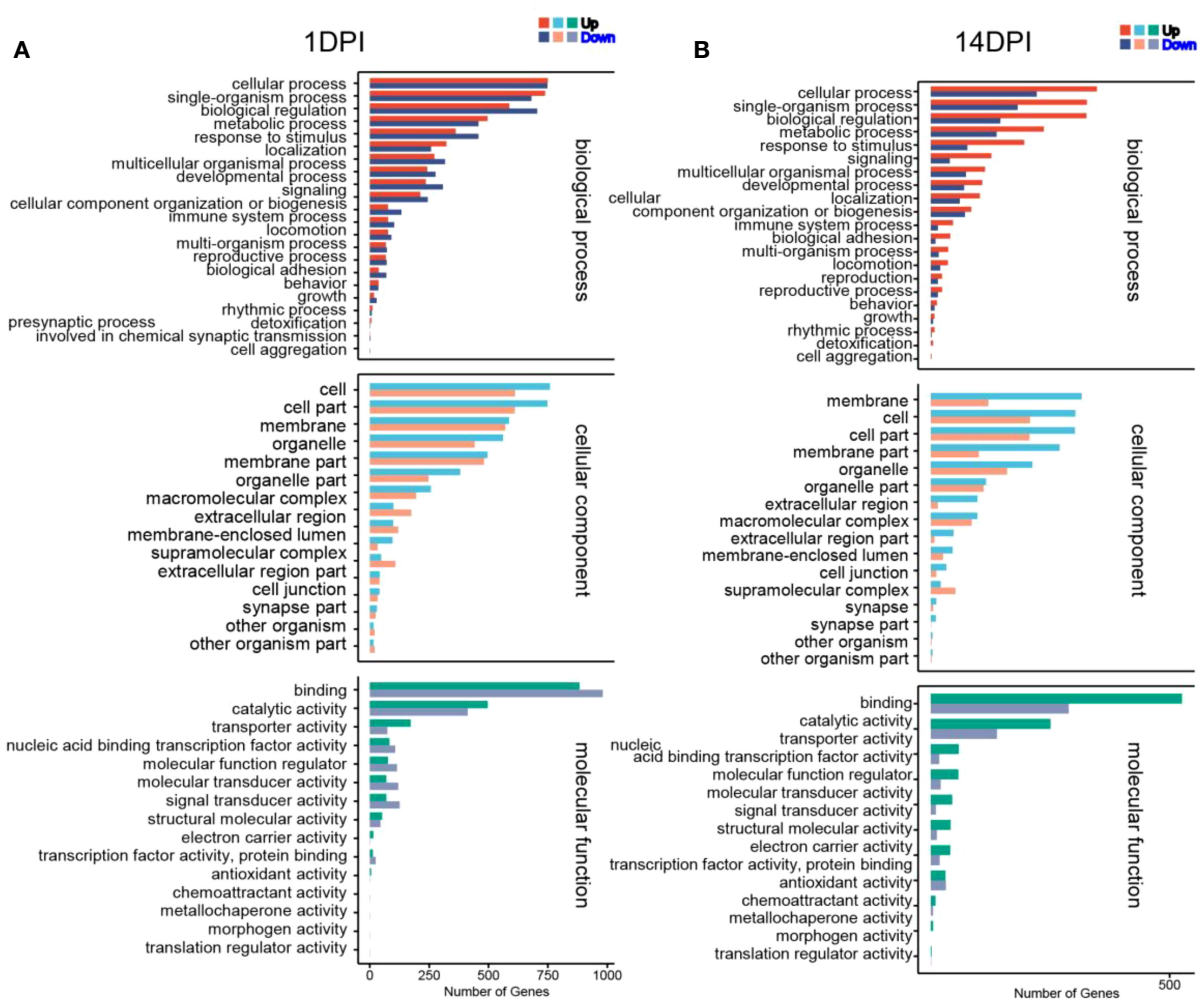
Transcriptomic analysis of change genes on *F. columnare*-stimulation in the ocular mucosa (OM) of rainbow trout using Gene Ontology enrichment analysis. **(A)** *F. columnare*-infected group vs. control group at 1 day post-infection (DPI). **(B)** *F. columnare*-infected group vs. control group at 14 DPI. The y-axis represents the Gene Ontology process, and the x-axis represents the number of genes in the process.

**Figure 5:**
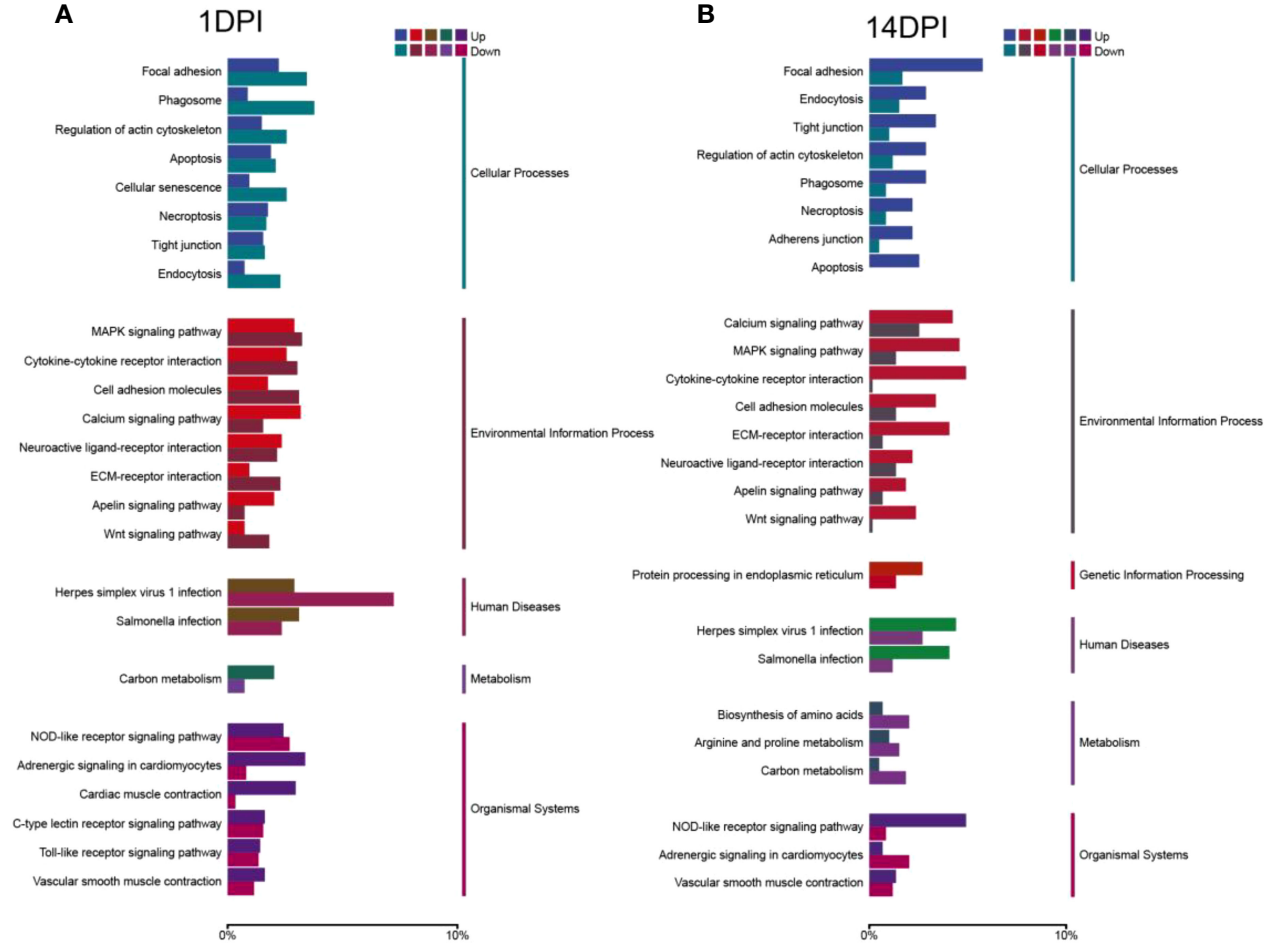
KEGG enrichment analysis of *F. columnare* stimulation on the transcriptomic changes in the ocular mucosa (OM) of trout. **(A)** *F. columnare*-infected group vs. control group at 1 day post-infection (DPI). **(B)** *F. columnare*-infected group vs. control group at 14 DPI. Genes are assigned to six special KEGG pathways, including cellular processes, environmental information processing, genetic information processing, human disease, metabolism, and organismal systems.

We then annotated the screened differential genes using different databases. A total of 4,255 DEGs were annotated, and the results of each database annotation are shown in **Supplementary Table S3**. Moreover, 3,447 DEGs had GO annotation and were classified into 52 and 48 functional items, and 3,459 DEGs had KEGG annotation and were classified into 25 functional items at 1 and 14 DPI, respectively (**Supplementary Table S4**). The ontology (GO) enrichment analysis revealed the top three enriched pathways within the “biological process,” “cellular component,” and “molecular function” categories (biological process: cellular process and single-organism process; cellular component: cell, cell part, and membrane; molecular function: binding, catalytic activity, and transporter activity) (**Figures 4A, B**). Additionally, the KEGG analyses revealed that the DEGs were predominantly associated with cellular processes and environmental information processing. Furthermore, compared to the 14 DPI, the DEGs at 1 DPI exhibited more pronounced enrichment responses in pathways including apoptosis, cytokine–cytokine receptor interaction, and Toll-like receptor signaling pathways (**Figures 5A, B**). These results indicate that a large number of immune-related signaling pathways are activated after *F. columnare* infection in the OM.

### Analysis of immune-related process in the transcriptome

**Figure 6:**
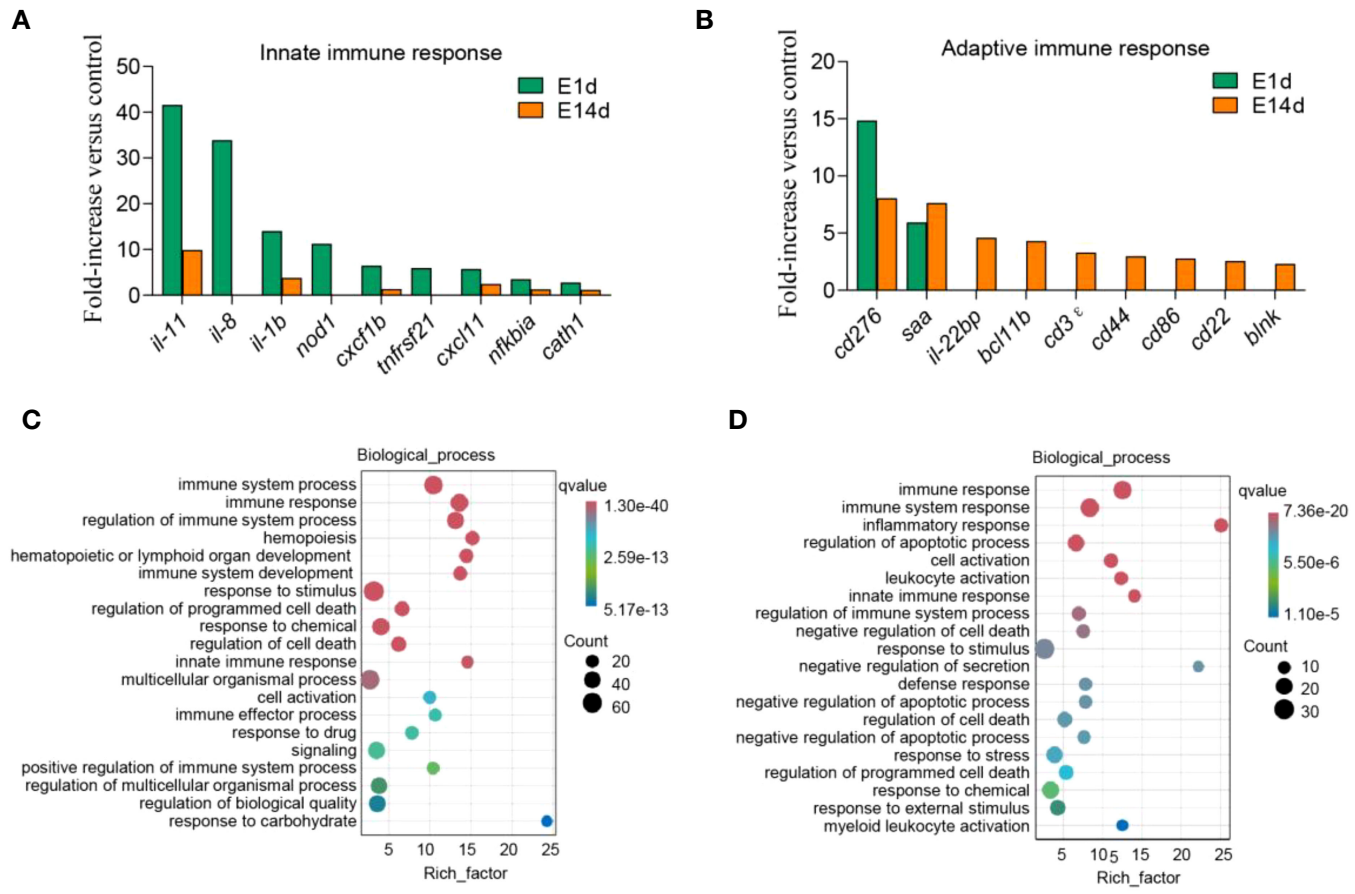
Upregulated immune genes and biological process enrichment analysis. **(A, B)** Representative innate **(A)** and adaptive **(B)** immune genes modulated by *F. columnare* infection at 1 and 14 days post-infection (DPI). Data are expressed as mean fold increase in expression. **(C, D)** Gene Ontology enrichment analysis of biological processes related to signal transduction, host defense responses, and immune responses. The left image shows 1 DPI **(C)**, and the right image shows 14 DPI **(D)**. The size of the bubbles represents the number of genes in a pathway, while the color represents the *q*-value.

**Figure 7:**
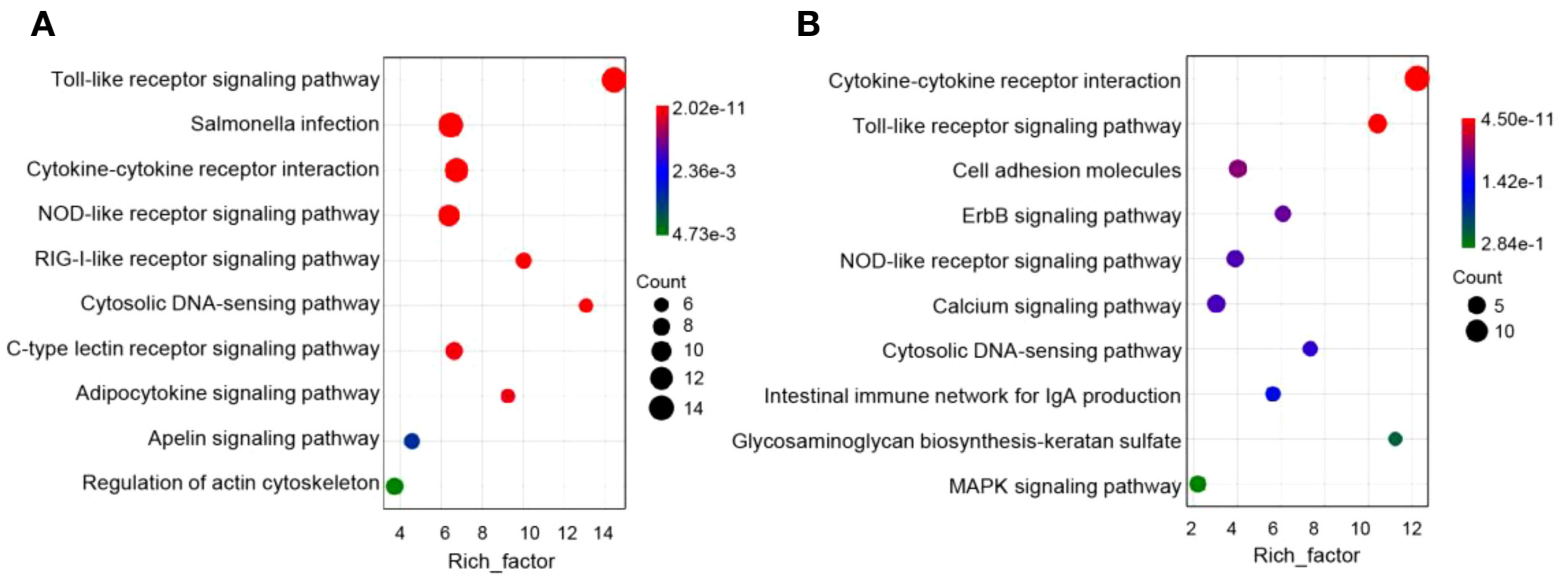
Pathway enrichment analysis of the upregulation of immune genes in rainbow trout infected with *F. columnare*. **(A)** Pathway enrichment analysis at 1 day post-infection (DPI). **(B)** Pathway enrichment analysis at 14 DPI.

Next, immune genes screened out from 1- and 14-DPI groups were analyzed by using the GO and KEGG databases (**Figures 6A–D**). The results showed that there were significant differences in the immune processes between 1 and 14 DPI. At 1 DPI, the GO analysis shows that a large number of the genes were enriched in the immune system process, immune process, and regulation of immune system process. Accordingly, a large number of genes related to innate immunity are upregulated, such as *il-11*, *il-8*, *il-1b*, *nod1*, and *cxcf1b*, whereas immune response, immune system process, and inflammatory response were enriched at 14 DPI. Genes associated with acquired immunity were upregulated such as *cd 276*, *saa*, *il-22bp*, *bcl11b*, *cd3ϵ*, *cd44*, *cd86*, etc. This suggests that two different immune response processes may occur in the early and late stages of infection. We further performed a KEGG analysis of upregulated differential genes at two time points (**Figures 7A, B**). The upregulated immune genes are mainly annotated in pattern recognition receptor-related signaling pathways like Toll-like, NOD-like, RIG-I-like, and cytosolic DNA-sensing receptor signaling pathways at 1 DPI. The OM recognizes bacteria through pattern recognition receptors to activate the innate immune response of the OM to clear the invading pathogens. Importantly, at 14 DPI, besides pattern recognition receptor-related signaling pathways being activated, the cell adhesion molecules and intestinal immune network for IgA production were also upregulated, implying that B/T and other lymphocytes were involved in the anti-bacterial immune process at a later period.

### Inflammation and negative effects on eye function after *F. columnare* infection

**Figure 8:**
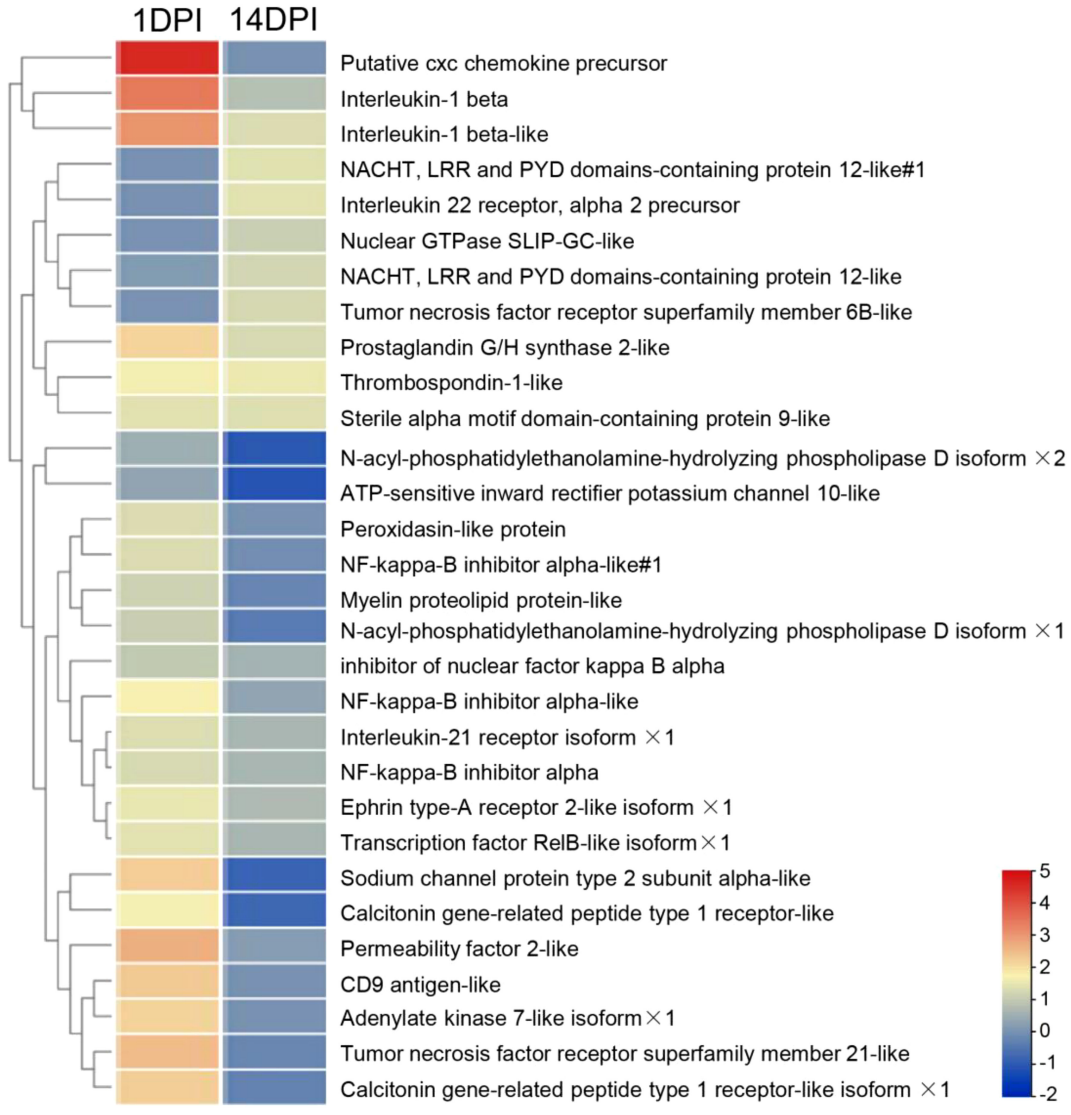
Heat map and cluster analysis showing the average classification composition of the top 30 differentially expressed inflammatory genes in groups at 1 and 14 days post-infection. The color represents the relative abundance of the corresponding genus, with red indicating the higher abundance and blue indicating the lower abundance. Pearson correlation was carried out, and the “complete” method was used to cluster the values.

**Figure 9:**
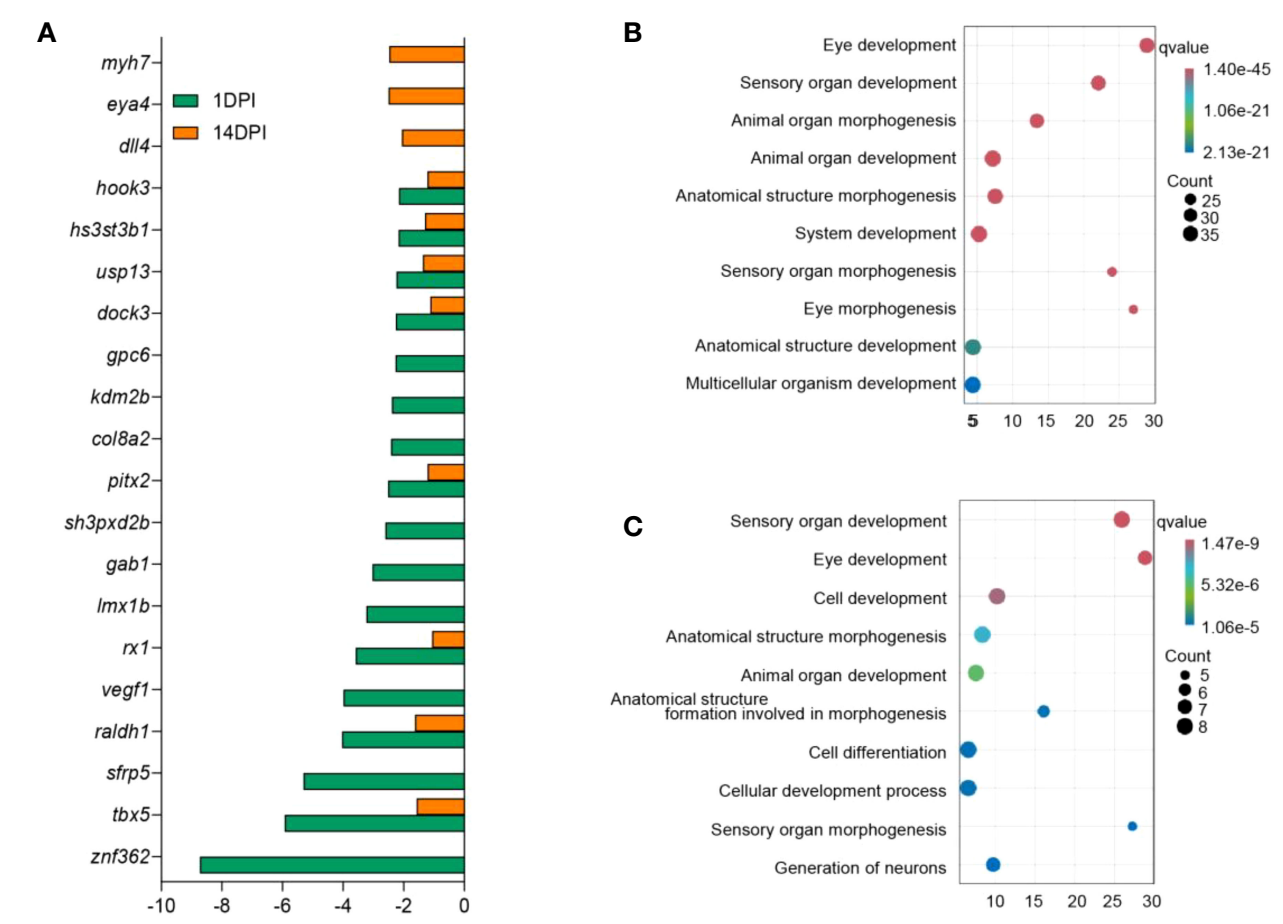
Analysis of differentially expressed genes and biological function enrichment in the ocular mucosa (OM) of trout after *F. columnare* infection. **(A)** Expression fold changes of eye function-related genes at 1 and 14 days post-infection (DPI). **(B, C)** Gene Ontology enrichment analysis of downregulated eye function-related genes in the OM of trout at 1 DPI **(B)** and 14 DPI **(C)**.

In mammals, inflammation induced by pathogens could injure the epithelium and adnexal structures of OM (31, 32). To understand the interaction between inflammation and eye function, we further analyzed the differentially expressed genes between the two. The top 30 differentially expressed inflammatory genes in groups at 1 and 14 DPI were screened, and the average classification compositions were posed through a heat map and cluster analysis (**Figure 8**). The results showed that “putative cxc chemokine precursor”, “interleukin-1 beta”, “sodium channel protein type 2 subunit alpha-like”, “permeability factor 2-like”, and “tumor necrosis factor receptor superfamily member 21-like” were significantly downregulated at 14 DPI. To explore the effect of mucosal immune response on eye function, we analyzed downregulated differentially expressed genes related to eye function. The results showed that *znf362*, *tbx5*, *sfrp5*, and *raldh1* were downregulated deeper at 1 DPI, while *myh7*, *eya4*, and *dll4* were downregulated deeper at 14 DPI (**Figure 9A**). The GO analysis result uncovered that eye development, sensory organ development, and animal organ morphogenesis were response processes. The group at 1 DPI had more intense changes (**Figures 9B, C**).

### Validation of RNA-seq results by qPCR

**Figure 10:**
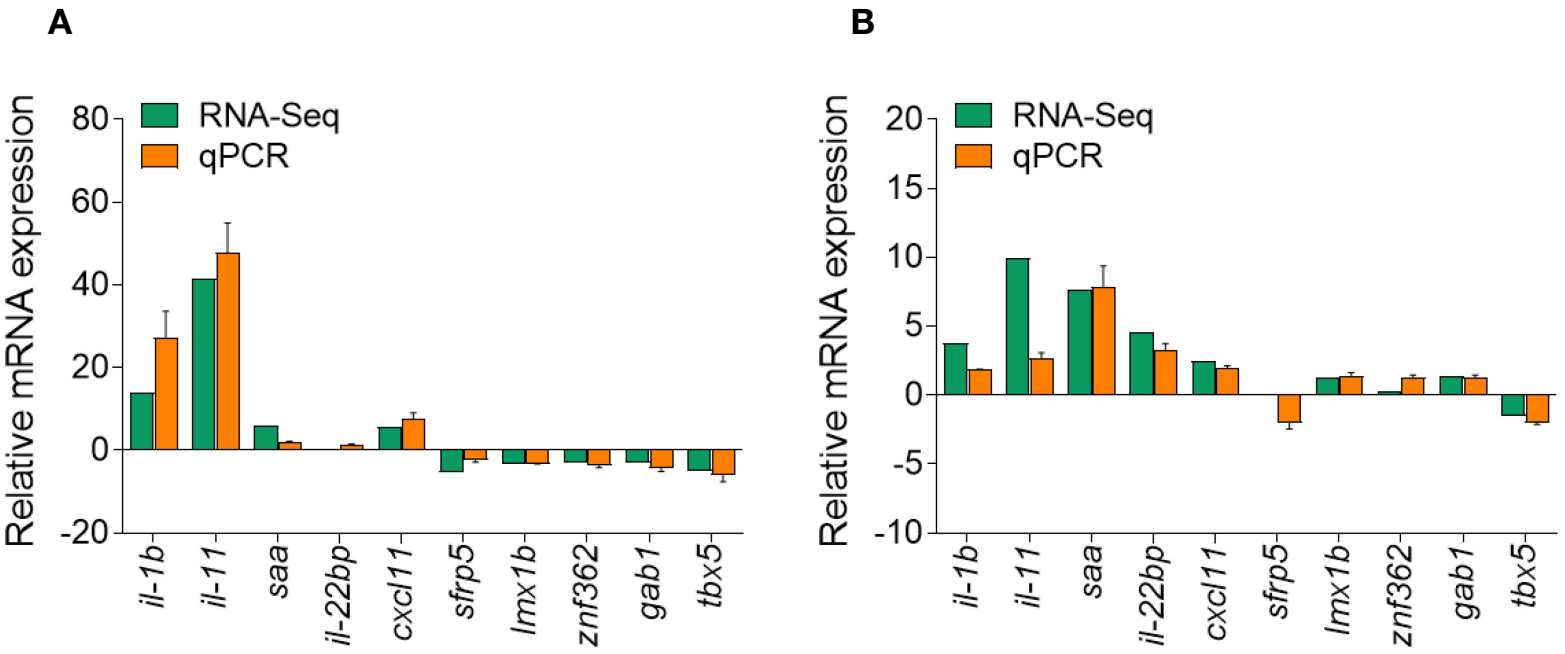
Comparison of the expression profiles of 10 selected genes as determined by RNA sequencing and qPCR. **(A)** Comparison of five upregulated genes and five downregulated genes at 1 days post-infection (DPI). **(B)** Comparison of five upregulated genes and five downregulated genes at 14 DPI. Data are representative of three independent experiments (mean ± SEM).

To validate the DEGs identified through RNA-seq analysis, 10 specific genes were selected for further evaluation *via* qPCR analysis, including five upregulated and five downregulated DEGs. The qPCR results showed significant and identical expression trends to those of the RNA sequencing data (**Figures 10A, B**). Therefore, the qPCR analysis results effectively validated the expressions of the DEGs identified through high-throughput sequencing analysis.

## Discussion

Fish live in an aquatic environment that, unlike air, is the ideal medium for microbial proliferation. Compared to terrestrial vertebrates, these conditions may present additional challenges to the OM immune system of aquatic vertebrates. Although the interaction between bacteria and some mucosal immune systems in teleost fish is well understood (21–24), the OM antibacterial immune process in teleost fish remains unknown. To our knowledge, this is the first study to display the OM transcriptome of rainbow trout after *F. columnare* infection.

Hence, to conduct an exploration of the immune responses of the OM in rainbow trout, we established an *F. columnare* infection model by immersion and analysis and detected immune-related gene expression. *F. columnare* can cause histological lesions of various mucosal tissues of rainbow trout and other teleost fish (33, 34), causing pronounced classical clinical symptoms including gill decay and fin erosion (18–20). Here we found that *F. columnare* could invade the OM, suggesting that trout OM could be a primary invasion site like skin and gill. Notably, *F. columnare* is mainly located in the epithelium of the OM, but in the olfactory organs the lamina propria was the main invasion location (22). These results hinted that ocular mucosal epithelial cells are the first line of defense against *F. columnare.* Importantly, our results found that the *F. columnare* intrusion triggered a strong immune response within the OM, characterized by the expression of an array of genes associated with immune regulation, including anti-bacterial, inflammatory, and immunoglobulin-related genes. Meanwhile, we found a strong correlation between the bacterial load invading the OM and the immune response. *igt* and *cxcl10* had differently longer and high expression levels in the OM compared to skin (21). The above-mentioned results indicate that the OM could mount a strong immune response to bacterial infection. To further understand the role of the OM against pathogens, transcriptome sequencing was performed on the samples at the above-mentioned two time points.

Using transcriptome sequencing, we next analyzed the overall gene expression in *F. columnare*-infected rainbow trout at 1 and 14 DPI. The transcriptome sequencing analysis highlighted the occurrence of an overall view of the biological processes in the trout OM following *F. columnare* infection. A total of 3,447 DEGs had GO annotation and were classified into 52 and 48 functional items, and 3,459 DEGs had KEGG annotation and were classified into 25 functional items at 1 and 14 DPI, respectively. The GO analysis results showed that the DEGs are mainly classified into cellular process, cell, and binding. Compared to 14 DPI, more DEGs were downregulated at 1 DPI, which also were detected in the transcriptome buccal and pharyngeal mucosa (22, 23). In mammals, homeostasis of the ocular surface is the result of an equilibrium regulated by a plethora of interacting entities (cells, extracellular matrix, nerves, oxygen supply, hormones, and cytokines, etc.) (35). We further performed KEGG analysis of the transcriptome to explore the efforts of OM to maintain mucosal homeostasis. KEGG results indicated that DEGs were classified into cellular processes (e.g. focal adhesion, phagosome), environmental information processing (e.g. cytokine-cytokine receptor interaction, MAPK signaling pathway), and organismal systems (NOD-like receptor signaling pathway, C-type lectin receptor signaling pathway). Immune-related signaling pathways made a great contribution, which was consistent with what we reported earlier in nasal, and pharyngeal mucosa (22, 23). Differently, bacterial invasion of mucosa could activate more pattern recognition receptors such as Toll-like, while Ich mainly activated NOD-like receptors (36, 37).

To better understand the immune process in trout OM upon *F. columnare* infection, we analyzed immune-related biological processes and signaling pathways following *F. columnare* infection. At 1 DPI, GO enrichment analysis indicated upregulated genes were classified into regulation of programmed cell death, regulation of cell death, and positive regulation of immune system process, whereas negative regulation of cell death, negative regulation of secretion, negative regulation of programmed cell death, and negative regulation of apoptotic process were active at 14 DPI. In mammals, as a reaction to external stimuli, the OM will temporarily shift its equilibrium, but return to homeostasis once the stimulus is absent (38). These results suggested that, like mammals, teleost ocular mucosal immunity plays a key role in maintaining ocular homeostasis through positive and negative regulation. Furthermore, innate immune response was invoked significantly at 1DPI, while adaptive immune related processes, such as, cell activation, leukocyte activation and myeloid leukocyte activation processes occurred at 14 DPI. These results suggested that innate and adaptive immunity play important roles in early and late stage after *F. columnare* infection, respectively. In our previous studies, *F. columnare* induced inflammation in the early stage of skin infection and induced IgT^+^/IgM^+^ B lymphocytes to play a clearing role in the later stage (21). Subsequently, we analyzed important molecules of innate and adaptive immunity in the OM at 1 and 14 DPI. Accordingly, *il-11*, *il-8*, and *il-1b* had high expression levels at 1 DPI. In mammals, *il-11* is produced by lung epithelial cells and immune cells which may contribute to disease pathogenesis (39, 40). *il-8* is a potent neutrophil chemotactic factor and a crucial mediator in neutrophil-dependent inflammation (41, 42). Moreover, we can observe a large number of immune cell markers upregulated at 14 DPI, such as *cd3ϵ*, *cd44*, *cd86*, and *cd22*. The KEGG analysis results suggested that pattern recognition receptor and IgA production signaling pathways were activated at 1 and 14 DPI. In teleost fish, the specific IgT found is similar to mammalian IgA and plays an important role in mucosal tissue (43, 44). In our previous study, we observed elevated levels of IgT concentration and higher bacteria-specific titers in the gills and skin following *F. columnare* infection, in comparison to other immunoglobulins (21, 24). Therefore, future studies need to identify the role of IgT to clear the pathogenic bacteria.

Studies have shown that the OM would lose its immune homeostasis after pathogen infection and presents variable degrees of inflammation, which lead to the impairment of eye function (45–47). In this study, we observed a large number of high expression inflammatory factors during *F. columnare* invasion like *cxc* chemokine, *il-1b*, permeability factor 2-like, and sodium channel protein, and 1 DPI had a stronger immune response intensity, while 14 DPI had more inflammatory genes with weaker up-regulation, such as *il-1b*, *il-22* receptor, and *tnfr6b*-like. Inflammation is an essential regulator of epithelial wound healing, but excessive inflammation can disrupt eye homeostasis (48, 49). In mammals, high *il-1b* levels result in increasing Th17-dominant immunopathology, and *il-1b* expression was limited to macrophages and neutrophils. To learn the destructive effect of the inflammatory microenvironment on ocular mucosal tissue, the down-regulation genes of ocular function were analyzed (50). Consistent with the expression levels of inflammatory factors, more genes were downregulated at 1 DPI (*znf632*, *tbx5*, *sfrp5*, and *vegf1*). However, these usually also play more specific roles in a wide variety of regulated biological processes, including signal transduction, cell growth, differentiation, and development (51–54). We further carried out GO analysis on these downregulated genes, and results showed that eye development, sensory organ development, animal organ morphogenesis, and cell development were primarily adversely affected. These results indicated that the inflammatory response occurred in trout OM might have an adverse effect on the eye visual function, thus the specific mechanism needs to be further studied.

In conclusion, our results, for the first time, explore the antibacterial immune response of trout OM. Here, we found that the *F. columnare* could successfully invade the trout OM, which is accompanied by tissue damage. Concurrently, *F. columnare* infection elicited a strong immune response of trout OM, characterized by the upregulation of innate and adaptive immunity-related genes, especially inflammatory cytokines and IgT. Importantly, our results emphasized that OM is an important component of the mucosal immune system in trout, and it may contribute to the research of mucosal immunity. However, certain aspects warrant further investigation, particularly in the regulatory mechanisms underlying the immune responses of the teleost OM.

## Data availability statement

The raw RNA sequencing data have been deposited in the NCBI Sequence Read Archive under BioProject accession number PRJNA1012574.

## Ethics statement

The animal study was reviewed and approved by the Animal Experiment Committee of Institute of Hydrobiology, Chinese Academy of Sciences. The study was conducted in accordance with the local legislation and institutional requirements.

## Author contributions

WK: Data curation, Formal analysis, Investigation, Writing – original draft. PY: Data curation, Formal analysis, Investigation, Writing – original draft. GD: Data curation, Formal analysis, Investigation, Writing – original draft. GC: Data curation, Formal analysis, Investigation, Writing – original draft. ZX: Funding acquisition, Project administration, Supervision, Writing – review & editing.
