# Supplementary material for: Elucidating the dynamic immune responses within the ocular mucosa of rainbow trout (Oncorhynchus mykiss) after infection with Flavobacterium columnare
Source: Front Immunol. 2023 Nov 21;14:1288223. doi: 10.3389/fimmu.2023.1288223 (PMC10702956; doi:10.3389/fimmu.2023.1288223)
Supplement: Supplementary file 1 [file Table_1.docx]

**Elucidating the dynamic immune responses within the ocular mucosa of rainbow trout (*Oncorhynchus mykiss*) after infection with *Flavobacterium columnare***

Weiguang Kong^1^, Peng Yang^1^, Guangyi Ding^1^, Gaofeng Cheng^2^, and Zhen Xu^1*^

^1^Key Laboratory of Breeding Biotechnology and Sustainable Aquaculture, Institute of Hydrobiology, Chinese Academy of Sciences, Wuhan 430072, China

^2^Department of Aquatic Animal Medicine, College of Fisheries, Huazhong Agricultural University, Wuhan, Hubei 430070, China

^*^Correspondence: Zhen Xu ([zhenxu@ihb.ac.cn](mailto:zhenxu@ihb.ac.cn))

**Supplementary materials**

**TABLE S1 |** Primers sequence list for qPCR.

| Gene | Primer Sequence (5’-3’) | | Product size |
| --- | --- | --- | --- |
|  | Forward primer | Reverse primer |  |
| *ef1α* | GGCAAGCCCATGTGTGTGGA | ACCACCCGAGGGACATCCTG | 199 |
| *igt* | CAGACAACAGCACCTCACCTA | GAGTCAATAAGAAGACACAACGA | 114 |
| *igm* | AAGAAAGCCTACAAGAGGGAGA | CGTCAACAAGCCAAGCCACTA | 156 |
| *pigr* | GTACAGCAGGTGTTCACAGTAAC | CCACAGACGACCTTGGATAAC | 145 |
| *cd86* | AGACGAGGGACCTACCATCC | CGTACAGCCCCTCATCTGAC | 114 |
| *c7-1* | TATCTTCACTGCCACGGTC | TAGCCTGTAACTCCACATAGAC | 218 |
| *il-6* | AAGACTCTTTGCTCCGCCTC | ATCAGCACAGTGGTCTGGTG | 152 |
| *il-8* | TGTCGTTGTGCTCCTGG | CCTGACCGCTCTTGCTC | 210 |
| *il-11* | CAGAGCGTCAAGGAAACAC | GCTCCTGGGAAGACTGTAA | 191 |
| *cd22* | GGCTGTGGGAAGTCGAATGA | TTCAAGTCCATCGTGGAGCC | 186 |
| *cd209* | GCCCTCTTTGTGCTGATT | ACTCCCTACACTTCCTTA | 214 |
| *saa* | TTGTTCTGACCCTCGTTG | CCTGGCAGCATCATAGTT | 167 |
| *ccl13* | CAGAACAACCTCCAGTAGC | ATCGTCGTCTTGGCAGTA | 147 |
| *mhc Ⅱ* | AATGGCGACTGGCACTA | GCCCGATGGCTATCTTA | 164 |
| *irf7* | GATGCCTCAAAATGAAATGG | TCAGGGAACTTCTCACCAAA | 131 |
| *nox1* | CACCAATCCACCACATAA | AGGAAGACATACTGACCC | 278 |
| *cxcl10* | ACATCAACGGTCCTCATC | ACACTTCTTCCCTTCTCC | 198 |
| *irg1* | CAGAGGTACTACAGGAAATGG | TTACTGGTCTTCAAGCAATG | 101 |
| *nos2* | GGCAGTCAAGAACCAACC | GAGCACCAAACGCTAATT | 253 |
| *nod1* | CCTGCTTTTCAAATACAAC | AGCCTGGATCTCATCATAG | 120 |
| *clec4e* | GCAGCCACCTTACCATC | CACCCATCTCCAATCCC | 136 |
| *nekb1* | ATGAAAACGGAGACACGCCT | AGGTGGTTGAGCTTGTCGAG | 123 |
| *tnfr2* | AGCAGAGTCGGATGTGGAA | GGTGGTTTGGAGGATGGAG | 195 |
| *prdm1* | GGCAAGGAGGACCAGAG | CCGGTTGTAGAGGCGTA | 143 |
| *il-1b* | TGATGAATGAGGCTATGGA | GATGGTGAAGGTGGTAAGG | 396 |
| *il-21r* | CTGAGCAAGAGCGGTCAAGA | TGGGAGGATTCCCCCTCTTC | 107 |
| *cxcl11* | GATGGCCTTCGCACCAAAAG | GGTTCCTGGGCATTTGCATC | 114 |
| *sfrp5* | TAAAGCCTGCCCTCCATGTG | TTCTGGGCAGCGATGAGTTT | 140 |
| *lmx1b* | TCAGTGTGCAACAAGGAGGG | TGATGGCATTCAGCACCGAT | 221 |
| *znf362* | CAGAAGCGAACAAAGCGACC | TGGTCTGATGTCCCGATTGC | 103 |
| *gab1* | CACCAGAACCTTCACCCGAG | ATTGTCATCTGGGTCGTCCG | 111 |
| *tbx5* | CAGTCTGGCTCCTACCCTCT | CGGGACGGTAGTAATGGTCG | 153 |
| *F. columnare*-  16 sRNA | GCCCAGAGAAATTTGGAT | GCCCA GAGAAATTTGGAT | 1193 |
| *F. columnare*-Abs | CCTGTACCTAATTGGGGAAAAGAGG | GTTGTATACACATCCGAAGTTCCAT | 202 |
| *F. columnare*-probe | FAM-ACAACAATGATTTTGCAGGAGGAGTATCTGATGGG-TAMRA | |  |

**TABLE S2 |** Statistical output of sequencing data.

| **Samples** | **Clean reads** | **Clean base** | **GC content** | **%≥Q30** |
| --- | --- | --- | --- | --- |
| FCC14d1 | 25,859,149 | 7,757,744,700 | 51.23% | 90.75% |
| FCC14d2 | 23,429,093 | 7,028,727,900 | 51.56% | 90.49% |
| FCC14d3 | 23,359,371 | 7,007,811,300 | 51.54% | 90.96% |
| FCC1d1 | 23,003,715 | 6,901,114,500 | 51.30% | 90.50% |
| FCC1d2 | 23,284,557 | 6,985,367,100 | 51.33% | 91.54% |
| FCC1d3 | 23,080,354 | 6,924,106,200 | 51.26% | 91.41% |
| FCE14d1 | 26,665,515 | 7,999,654,500 | 51.56% | 91.12% |
| FCE14d2 | 24,965,363 | 7,489,608,900 | 51.65% | 90.97% |
| FCE14d3 | 25,529,984 | 7,658,995,200 | 51.63% | 90.80% |
| FCE1d1 | 24,500,947 | 7,350,284,100 | 51.24% | 91.20% |
| FCE1d2 | 22,305,789 | 6,691,736,700 | 51.29% | 90.99% |
| FCE1d3 | 27,089,327 | 8,126,798,100 | 51.18% | 91.16% |

**TABLE S3 |** Reads mapping information.

| **Sample** | **Total Reads** | **Mapped Reads** | **Uniq Mapped Reads** | **Multiple Map Reads** |
| --- | --- | --- | --- | --- |
| FCC14d1 | 51,718,298 | 37,938,717 (73.36%) | 34,816,433 (67.32%) | 3,122,284 (6.04%) |
| FCC14d2 | 46,858,186 | 34,857,889 (74.39%) | 31,206,020 (66.60%) | 3,651,869 (7.79%) |
| FCC14d3 | 46,718,742 | 34,719,230 (74.32%) | 31,252,505 (66.90%) | 3,466,725 (7.42%) |
| FCC1d1 | 46,007,430 | 33,365,280 (72.52%) | 30,629,104 (66.57%) | 2,736,176 (5.95%) |
| FCC1d2 | 46,569,114 | 34,402,653 (73.87%) | 31,463,688 (67.56%) | 2,938,965 (6.31%) |
| FCC1d3 | 46,160,708 | 33,448,959 (72.46%) | 30,731,900 (66.58%) | 2,717,059 (5.89%) |
| FCE14d1 | 53,331,030 | 38,757,616 (72.67%) | 35,820,382 (67.17%) | 2,937,234 (5.51%) |
| FCE14d2 | 49,930,726 | 36,737,239 (73.58%) | 33,814,865 (67.72%) | 2,922,374 (5.85%) |
| FCE14d3 | 51,059,968 | 36,653,134 (71.78%) | 33,617,755 (65.84%) | 3,035,379 (5.94%) |
| FCE1d1 | 49,001,894 | 36,496,172 (74.48%) | 32,864,819 (67.07%) | 3,631,353 (7.41%) |
| FCE1d2 | 44,611,578 | 33,507,123 (75.11%) | 30,203,744 (67.70%) | 3,303,379 (7.40%) |
| FCE1d3 | 54,178,654 | 40,503,752 (74.76%) | 36,314,213 (67.03%) | 4,189,539 (7.73%) |

**TABLE S4 |** Functional annotation and enrichment analysis of DEGs.

| **DEG Set** | **Total** | **COG** | **GO** | **KEGG** | **KOG** | **NR** | **Pfam** | **Swiss-Prot** | **eggNOG** |
| --- | --- | --- | --- | --- | --- | --- | --- | --- | --- |
| FCC14d vs FCE14d | 1,099 | 192 | 868 | 898 | 581 | 1,071 | 860 | 596 | 995 |
| FCC1d vs FCE1d1 | 3,156 | 559 | 2,579 | 2,561 | 1,696 | 3,070 | 2,535 | 1,713 | 2,919 |
